# Relationship between neck circumference and body composition: a cross-sectional study based on a Chinese population

**DOI:** 10.3389/fpubh.2025.1693065

**Published:** 2025-11-07

**Authors:** Meiling Hu, Min Zhou, Xin Sun, Manfen Qing, Minxia Jiang

**Affiliations:** 1 The Second Affiliated Hospital of Nanchang University, Nanchang, China; 2 School of Nursing, Jiangxi Medical College, Nanchang University, Nanchang, China

**Keywords:** neck circumference, obesity, fat mass index, body composition, correlation

## Abstract

**Background:**

Obesity is a chronic metabolic disorder characterized by excessive fat accumulation, physiological dysfunction, and metabolic disturbances, closely associated with diabetes, cardiovascular diseases, and dyslipidemia. Currently, the primary indices for obesity screening include Body Mass Index (BMI), Waist Circumference (WC), and Percentage of Body Fat (PBF). However, these indices have certain limitations in practical application. Neck Circumference (NC), a novel anthropometric index, has garnered increasing attention in recent years.

**Objective:**

This study aims to assess the correlation between NC and several obesity-related metrics, including BMI, PBF, Fat Mass Index (FMI), and Visceral Fat Area (VFA). Furthermore, the reliability of NC as a diagnostic tool for obesity is evaluated.

**Methods:**

This study retrospectively analyzed body composition data from 8,319 Chinese young adults. For statistical analyses, IBM SPSS Statistics version 26.0 and R version 4.5.1 were used, which included multi-model fitting for verifying linear correlations, followed by Pearson correlation analysis, gender-stratified linear regression analysis, Receiver Operating Characteristic (ROC) curve analysis, calibration curve analysis, and Decision Curve Analysis (DCA). The optimal cut-off value of NC was determined using the Youden index, and its consistency with BMI-defined classification of obesity was validated using the Kappa test.

**Results:**

NC was significantly positively correlated with BMI, FMI, VFA, PBF (all *p* < 0.01), and its obesity diagnostic performance differed by gender. In females, NC’s AUC was 0.982 (vs. VFA 0.976), optimal cut-off 36.25 cm. Males had lower NC AUC (0.918) than BMI (0.979), PBF (0.985), VFA (0.954), optimal cut-off 38.95 cm (all *p* < 0.001). The female model showed better calibration (Brier = 0.0259 vs. 0.0648 in males). DCA confirmed NC’s obesity clinical utility across wide thresholds. Kappa testing revealed good consistency in obesity classification between NC and BMI (all *p* < 0.001), with higher Kappa in females (0.790) than males (0.644).

**Conclusion:**

NC correlates with key obesity indices. It is a simple, effective obesity screening tool for young females, while males require comprehensive assessment with BMI, PBF, and VFA.

## 1 Introduction

The epidemic of obesity has emerged as a global health crisis, transcending national borders and showing a consistent rise in prevalence across nearly all countries and regions worldwide. Projections indicate that by 2030, nearly 3 billion adults globally will be classified as overweight or obese, representing 50% of the total adult population at that time, with the number of people with obesity expected to reach 1.13 billion (1, 2). Obesity is associated with an increased risk of diabetes, cardiovascular diseases, cancer, osteoarthritis, sleep apnea, and depression (3). Furthermore, obesity can reduce life expectancy by approximately 5 to 20 years, thereby posing a serious challenge to public health systems (4–6). The economic losses associated with obesity are projected to reach $4.32 trillion, accounting for 2.9% of the global gross domestic product (1). Notably, the annual increase in obesity prevalence among adults in low- and middle-income countries in Asia is 2.1 times that in developed countries (7). At the same BMI, Asian adults exhibit a Percentage of Body Fat (PBF) that is 3–5% higher and a Visceral Fat Area (VFA) that is 15–20% greater in than Caucasian adults (8). This discrepancy contributes to a significantly elevated risk of diabetes and cardiovascular diseases among people with obesity in these populations.

Fortunately, appropriate treatment for people with obesity can reduce the incidence of various chronic non-communicable diseases and significantly enhance their quality of life. Therefore, early screening of people with obesity is both necessary and beneficial. Obesity can be assessed using indicators such as BMI and Waist Circumference (WC); however, these methods have limitations as they do not accurately reflect the distribution of body fat or distinguish between fat mass and fat-free mass. Consequently, their practical applications are constrained (9). WC serves as an indicator of abdominal fat accumulation; however, its measurement accuracy can be significantly influenced by factors such as respiration and dietary habits (10). For example, the use of WC as a diagnostic tool for central obesity is not applicable to pregnant individuals or people with ascites or other abdominal masses. This limitation occurs because of physiological changes in body composition during pregnancy and the potential for misinterpretation of WC measurements in the presence of excess fluid or tissue. Studies have demonstrated that Neck Circumference (NC) can serve as a straightforward anthropometric indicator for evaluating upper body fat accumulation. It exhibits characteristics of simple measurement, clear anatomical landmarks, minimal interference from external factors, high repeatability, and low variability. Therefore, NC specifically addresses the limitations of body mass index (BMI) and WC in terms of fat distribution assessment, measurement stability, and applicability to special populations, thereby functioning as a supplementary tool for obesity assessment (11).

In addition, NC has garnered significant attention as an indicator for assessing regional fat distribution. Neck adipose tissue is notably associated with cardiovascular risk factors, interleukin-7, leptin, and adiponectin (12). Furthermore, a larger volume of neck adipose tissue relative to height is directly correlated with long-term mortality (13). Increased NC is significantly associated with cardiovascular risk factors such as type 2 diabetes, hypertension, and dyslipidemia (14). Previous studies have confirmed a significant correlation between NC and BMI, WC, and VFA. In adulthood, body composition changes with age, while BMI may remain stable (15). Computed Tomography (CT), Magnetic Resonance Imaging (MRI), and Dual-energy X-ray Absorptiometry (DXA) are commonly used techniques for body composition assessment. However, due to issues such as radiation exposure, time-consuming procedures, and high testing costs, CT or MRI are difficult to adapt to routine clinical assessment scenarios. In contrast, Bioelectrical Impedance Analysis (BIA) offers advantages such as easy accessibility, affordability, and absence of radiation risk, making it a more suitable method for routine clinical assessment of body composition (16). Currently, only a limited number of countries have established NC thresholds based on their population data, and no uniform global standard has been established to date (17). As a populous country in Asia, China’s youth population serves as the primary reserve and active force of the labor market, as well as a high-risk group for obesity and related diseases (18, 19). However, there is still a lack of research on exclusive obesity screening indicators for this demographic, and there is an urgent need to establish assessment tools tailored to their body fat characteristics.

Therefore, this study aims to analyze the correlation between NC and body composition in a physical examination population of young people. This study provides a theoretical basis for NC to serve as an important indicator for the convenient screening of obesity, thereby supporting the early identification and precise prevention and control of obesity, which holds significant public health significance.

## 2 Methods

### 2.1 Participants

This study retrospectively analyzed data from young people who underwent physical examinations at the physical examination center of the Second Affiliated Hospital of Nanchang University from January 2022 to August 2024. The inclusion criteria were as follows: participants were required to have complete data, be aged 18 to 44 years, and be in a healthy physical condition. Exclusion criteria encompassed pregnant or lactating women and people who underwent repeated physical examinations (only the initial set of data was retained to eliminate redundancy). Sample size calculation for the correlation analysis was conducted using G*Power software. The procedure is outlined as follows: select ‘correlation: point biserial model’ as the statistical test method and input the relevant parameters. The effect size was set at 0.3, the significance level at 0.05, and the statistical power at 0.95. Based on these parameters, a minimum sample size of 109 individuals was determined. A total of 8,319 participants were included in the analysis, comprising 5,153 males and 3,166 females. This study received approval from the Ethics Committee of the Second Affiliated Hospital of Nanchang University (approval number: IIT-2024-303).

### 2.2 Data collection

All measurements were conducted in a dedicated testing room, and the measuring instruments were regularly calibrated and inspected. Neck circumference is measured using a soft tape measure. The subject should maintain a neutral posture, looking straight ahead with arms hanging naturally at their sides and feet together, while ensuring the neck is relaxed to minimize muscle tension. The soft tape is applied snugly against the skin, encircling the neck horizontally at the level of the lower edge of the spinous process of the seventh cervical vertebra and the lower edge of the thyroid cartilage at the front, or at the midpoint of the neck. The same measurer conducts two consecutive measurements. If the difference between the two measurements is ≤ 0.5 cm, the average is recorded. If the difference exceeds 0.5 cm, the measurement is repeated (20). NC is recorded in centimeters, rounded to one decimal place. Body weight, BMI, FMI, VFA, and PBF were measured using a body composition analyzer (InBody720, Bytimes, Korea). Prior to measurement, participants were instructed to fast for 4 h, empty their bladders, wear lightweight, metal-free clothing, and stand barefoot to ensure full contact of the soles of their feet with the electrode sheet. During the measurement, the arms were naturally lowered at an angle of 15° from the trunk, and participants were required to hold the electrode handle using the palms, thumbs, and fingers of both hands, remaining still for one minute to facilitate the measurement of body composition. Height was measured using a calibrated stadiometer to ensure accuracy. The criteria for obesity assessment were based on the reference standard for fat mass index, with people diagnosed as having obesity if their fat mass index (FMI) ≥ 9.0 kg/m^2^ in men and FMI ≥ 12.0 kg/m^2^ in women (21).

### 2.3 Statistical analysis

All data were independently entered into a newly Excel database by two graduate students from the research team. Data processing and analysis were performed using IBM SPSS Statistics (version 26.0) and R software version 4.5.1. If quantitative data followed a normal distribution, it was represented using the mean ± standard deviation, and a t-test was employed. If quantitative data did not follow a normal distribution, it was represented using the median (interquartile range), and the Mann–Whitney U-test was used. Categorical Data were represented as percentages (%) (22, 23). After confirming a linear relationship via multi-model fitting (linear, quadratic, logarithmic, and exponential models) and residual analysis, Pearson correlation analysis was used to explore correlations between NC and BMI, FMI, VFA, and PBF. Subsequently, linear regression analysis was performed with NC as the independent variable and each body composition index as the dependent variable, and the coefficient of determination (R^2^) was used to assess the extent to which NC explains their variation. The efficacy of NC in predicting obesity was evaluated from three dimensions: discriminative ability, calibration, and clinical utility. Discriminative ability was assessed using the area under the receiver operating characteristic curve (AUC); calibration curves were plotted to evaluate predictive accuracy; and decision curve analysis was conducted to assess clinical net benefit (24). The optimal cut-off value of NC for diagnosing obesity was determined based on the principle of maximizing the Youden index. The Youden index, a single statistical measure quantifying diagnostic test performance, is calculated, Youden index = sensitivity + specificity - 1 (25). The consistency of obesity classification between NC and BMI was analyzed using the Kappa test (26). All tests were two-sided; *p* < 0.05 was considered significant.

## 3 Results

### 3.1 General characteristics of the participants

This study included a total of 8,319 participants, comprising 61.94% (*N* = 5,153) males and 38.06% (*N* = 3,166) females. The obesity rates, diagnosed using FMI, were 14.01% in males and 7.23% in females. The average ages of males and females were 27.22 ± 3.81 years and 27.07 ± 3.91 years, respectively. Except for age, males exhibited higher values for height, weight, NC, BMI, and VFA compared to females. Conversely, FMI and PBF were lower in males, and these differences were statistically significant (*p* < 0.05). The average height was 172.37 cm (SD: 6.22) for males and 160.58 cm (SD: 5.68) for females. The average weight of male participants was 75.99 kg (SD: 13.05), compared to 59.33 kg (SD: 11.72) for female participants. The average NC for male participants was 37.64 cm (SD: 2.31), whereas for female participants, it was 33.17 cm (SD: 3.09). The average FMI for male participants was 6.51 kg/m^2^ (SD: 2.52), while female participants had an average FMI of 7.33 kg/m^2^ (SD: 3.08). The average BMI for male participants was 25.53 kg/m^2^ (SD: 3.84), in contrast to an average BMI of 23.00 kg/m^2^ (SD: 4.29) for female participants. The average PBF for male participants was 24.77% (SD: 6.13), while female participants had an average PBF of 30.81% (SD: 6.98). Finally, the VFA for male participants was 95.62 cm^2^ (SD: 6.13), whereas female participants had an average VFA of 73.26 cm^2^ (SD: 32.45). See Table 1 for detailed statistics.

**Table 1:** Characteristics of participants: Chinese young adults, 2022–2024.

Variable	Male (*n* = 5153)	Female (*n* = 3166)	All (*n* = 8319)	*p* value
Age (years)	27.22 ± 3.81	27.07 ± 3.91	27.17 ± 3.85	0.084
Height (cm)	172.37 ± 6.22	160.58 ± 5.68	167.88 ± 8.31	< 0.001
Weight (kg)	75.99 ± 13.05	59.33 ± 11.72	69.51 ± 14.94	< 0.001
NC (cm)	37.64 ± 2.31	33.17 ± 3.09	35.94 ± 3.41	< 0.001
FMI (kg/m^2^)	6.51 ± 2.52	7.33 ± 3.08	6.82 ± 2.77	< 0.001
BMI (kg/m^2^)	25.53 ± 3.84	23.00 ± 4.29	24.57 ± 4.20	< 0.001
PBF (%)	24.77 ± 6.13	30.81 ± 6.98	27.07 ± 7.10	< 0.001
VFA (cm^2^)	95.62 ± 30.44	73.26 ± 32.45	87.11. ± 33.05	< 0.001

NC, neck circumference; FMI, fat mass index; BMI, body mass index; PBF, percentage of body fat; VFA, visceral fat area.

### 3.2 Associations between neck circumference and body composition

**Figure 1:**
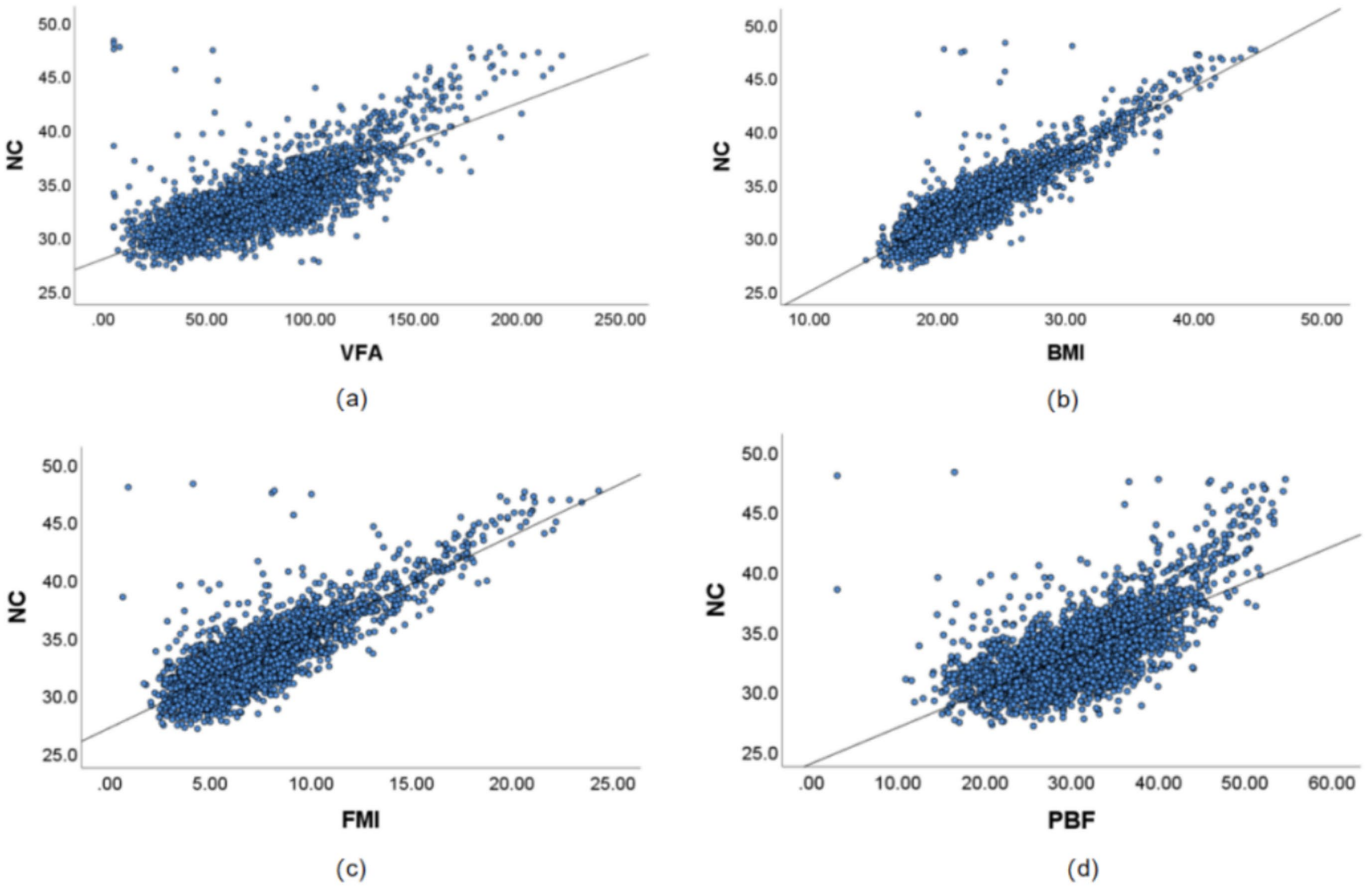
Correlation results between NC and VFA, PBF, FMI, and BMI in Chinese young adult females, 2022–2024. **(a)** Scatter plot of the correlation between NC and VFA, **(b)** Scatter plot of the correlation between NC and BMI, **(c)** Scatter plot of the correlation between NC and FMI, **(d)** Scatter plot of the correlation between NC and PBF. NC, neck circumference; FMI, fat mass index; BMI, body mass index; PBF, percentage of body fat; VFA, visceral fat area.

**Figure 2:**
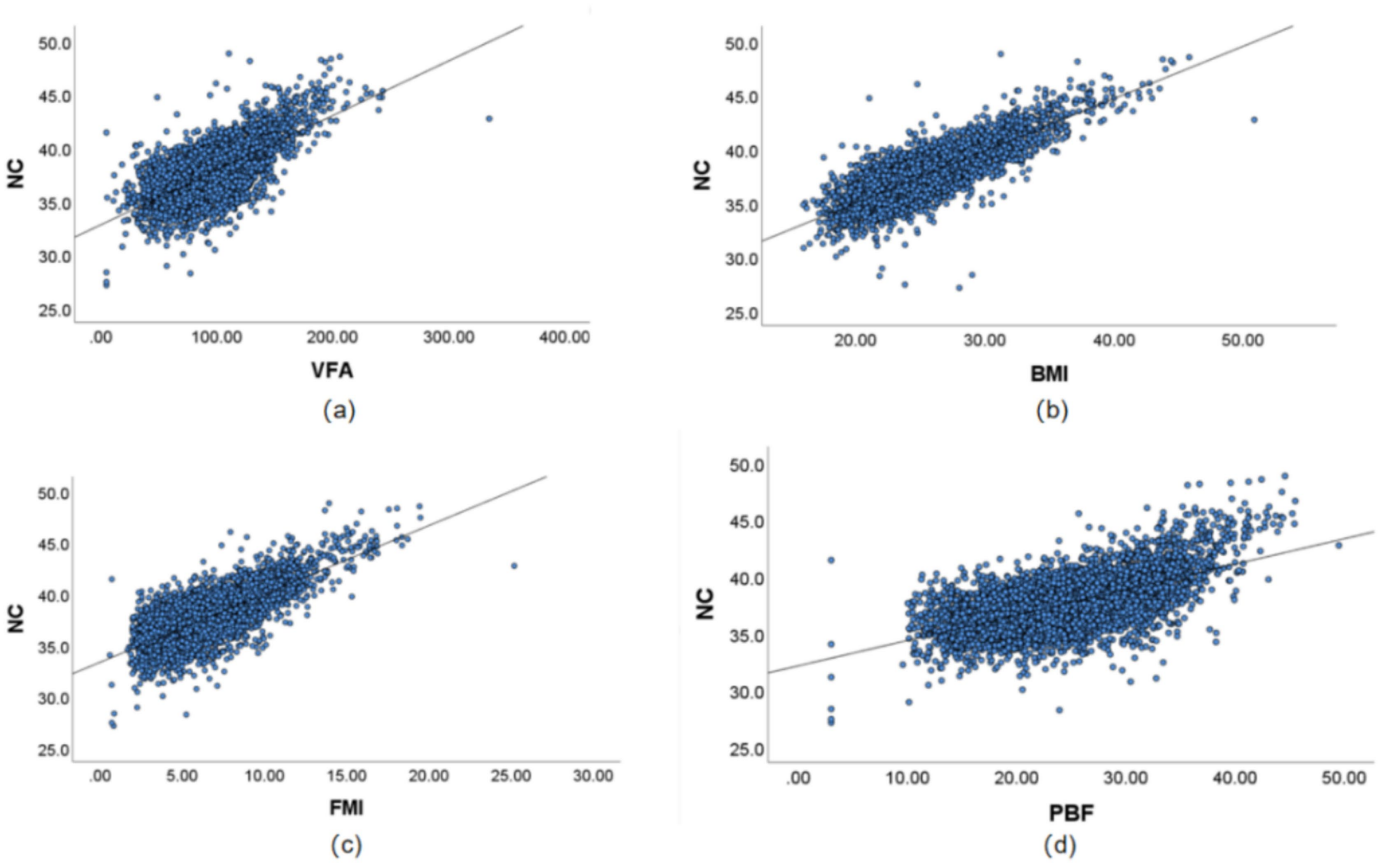
Correlation results between NC and VFA, PBF, FMI, and BMI in Chinese young adult males, 2022–2024. **(a)** Scatter plot of the correlation between NC and VFA. **(b)** Scatter plot of the correlation between NC and BMI. **(c)** Scatter plot of the correlation between NC and FMI. **(d)** Scatter plot of the correlation between NC and PBF. NC, neck circumference; FMI, Fat Mass Index; BMI, body mass index; PBF, percentage of body fat; VFA, visceral fat area.

Through multi-model fitting (linear, quadratic, logarithmic, and exponential models) to verify the type of relationship, the results showed that the association between NC and BMI, VFA, FMI, and PBF was predominantly linear. The results indicated that the associations of NC with BMI, VFA, FMI, and PBF were predominantly linear (Supplementary Tables S1–S4 present the comparison of the coefficient of determination *R*^2^ across multiple models; Supplementary Figures S1, S2 illustrate the residual normality tests and homoscedasticity tests). Consequently, Pearson correlation analysis was employed to quantify the strength of the linear associations between variables, and linear regression analysis was further conducted to assess the proportion of variance in the dependent variable explained by the independent variables. The pearson correlation coefficients between NC and various gender-stratified anthropometric indices, namely BMI, VFA, FMI, and PBF, showed significant positive correlations (*p* < 0.01). The correlations, listed in descending order of strength, were as follows: BMI (female 0.889, male 0.807), FMI (female 0.829, male 0.727), VFA (female 0.760, male 0.675), and PBF (female 0.683, male 0.594). The linear regression analysis results indicate that the proportion of variance in NC explained by each obesity parameter (*R*^2^) is consistent with the strength of their correlation. In the male population, BMI had the highest explanatory proportion (*R*^2^ = 0.652), while PBF was the lowest (*R*^2^ = 0.353). In the female population, BMI had the highest explanatory proportion (*R*^2^ = 0.790), and PBF was the lowest (*R*^2^ = 0.467). The fit of all regression models reached a significant level (*p* < 0.001). See Table 2 and Figures 1, 2 for visual representation.

**Table 2:** Sex-specific linear regression analysis of correlations between NC and body composition indices (BMI, FMI, VFA, PBF) in Chinese young adults, 2022–2024.

Sex	Dependent variable	Regression equation	Regression coefficient *b* (95% CI)	*r*	*R* ^2^	*F* value	*p* value
Male	BMI	BMI = −25.021 + 1.343 × NC	1.343 (1.316, 1.370)	0.807	0.652	9650.894	< 0.001
	FMI	FMI = −23.396 + 0.794 × NC	0.794 (0.774, 0.815)	0.727	0.529	5784.695	< 0.001
	VFA	VFA = −239.721 + 8.909 × NC	8.909 (8.644, 9.175)	0.675	0.456	4321.720	< 0.001
	PBF	PBF = −34.668 + 1.579 × NC	1.579 (1.521, 1.638)	0.594	0.353	2809.829	< 0.001
Female	BMI	BMI = −17.940 + 1.234 × NC	1.234 (1.212, 1.256)	0.889	0.790	11904.246	< 0.001
	FMI	FMI = −20.100 + 0.827 × NC	0.827 (0.808, 0.846)	0.829	0.688	6972.350	< 0.001
	VFA	VFA = −191.749 + 7.991 × NC	7.991 (7.752, 8.229)	0.760	0.577	4324.751	< 0.001
	PBF	PBF = −20.447 + 1.545 × NC	1.545 (1.488, 1.603)	0.683	0.467	2768.080	< 0.001

NC, neck circumference; FMI, fat mass index; BMI, body mass index; PBF, percentage of body fat; VFA, visceral fat area.

### 3.3 Gender differences and multidimensional performance evaluation of neck circumference in predicting obesity in young adults

**Figure 3:**
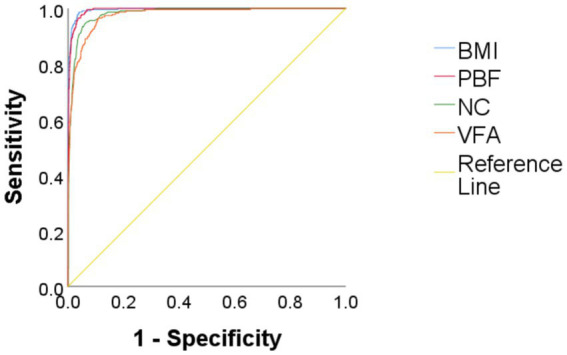
Comparison of ROC curves for predicting obesity using BMI, PBF, NC, and VFA in Chinese young adult females, 2022–2024. NC, neck circumference; FMI, fat mass index; BMI, body mass index; PBF, percentage of body fat; VFA, visceral fat area.

**Figure 4:**
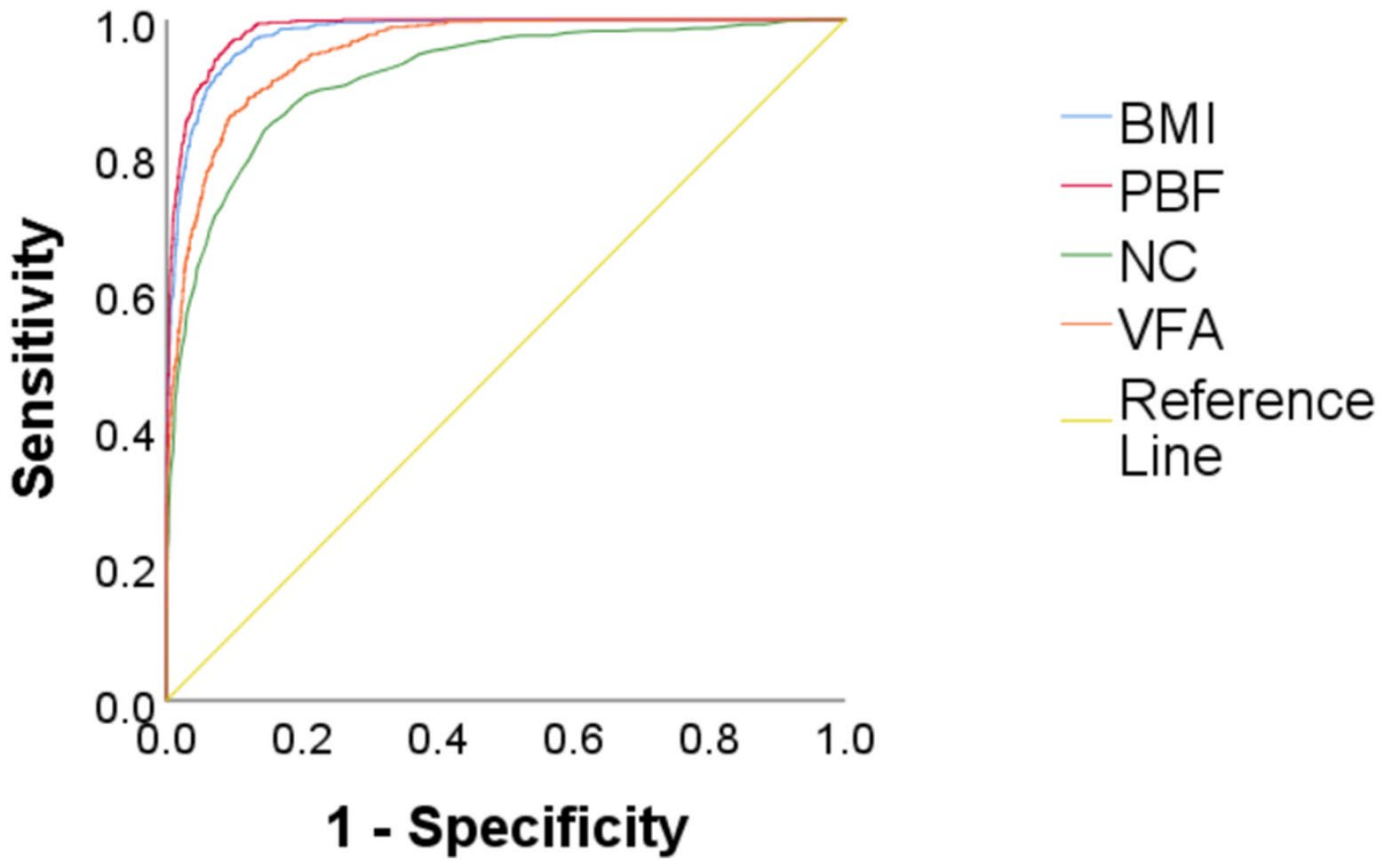
Comparison of ROC curves for predicting obesity using BMI, PBF, NC, and VFA in Chinese young adult males, 2022–2024. NC, neck circumference; FMI, fat mass index; BMI, body mass index; PBF, percentage of body fat; VFA, visceral fat area.

**Figure 5:**
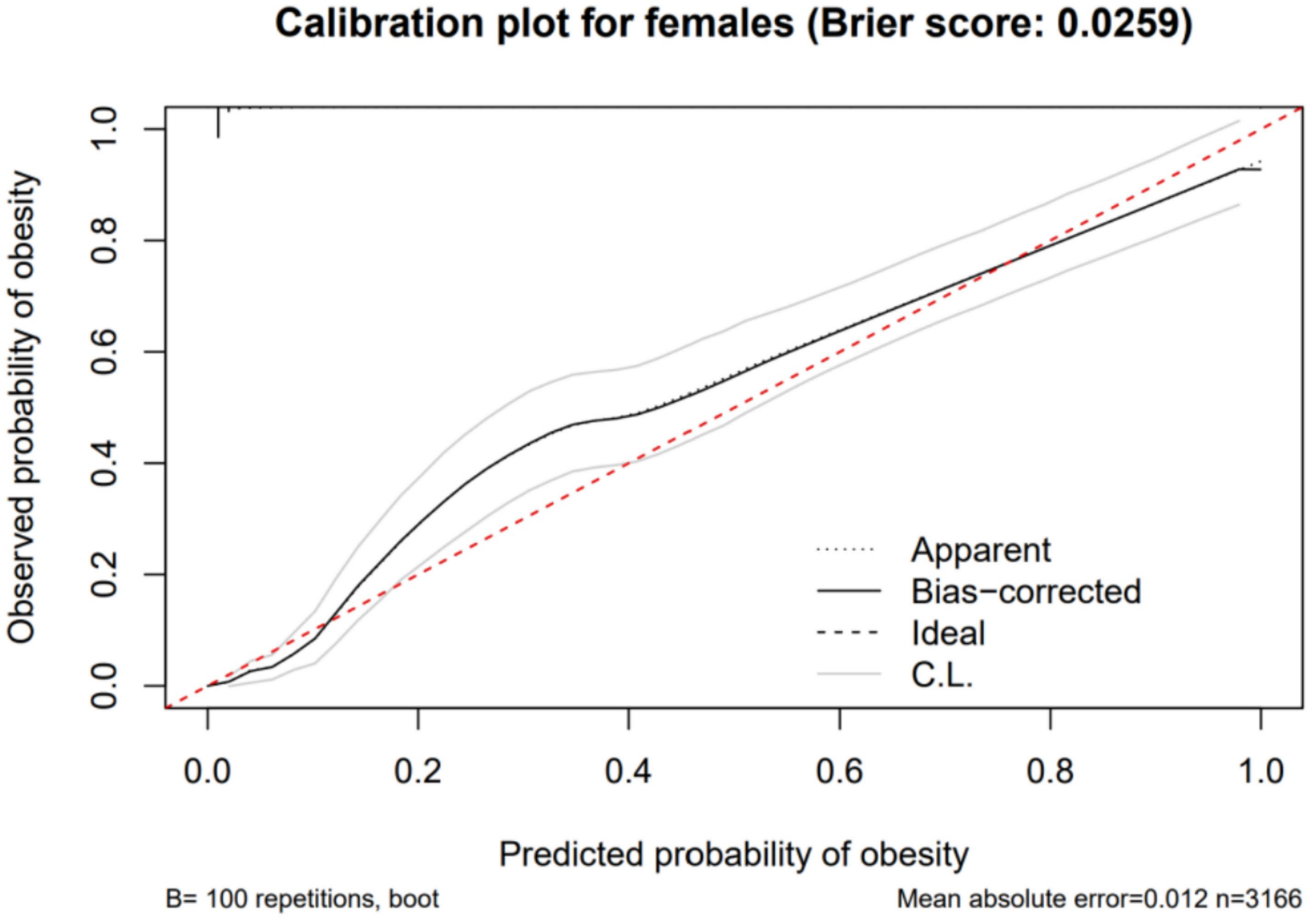
Calibration curve showing the expected rate of obesity predicted by neck circumference compared with the observed rate (with 95% CI) in Chinese young adult females, 2022–2024.

**Figure 6:**
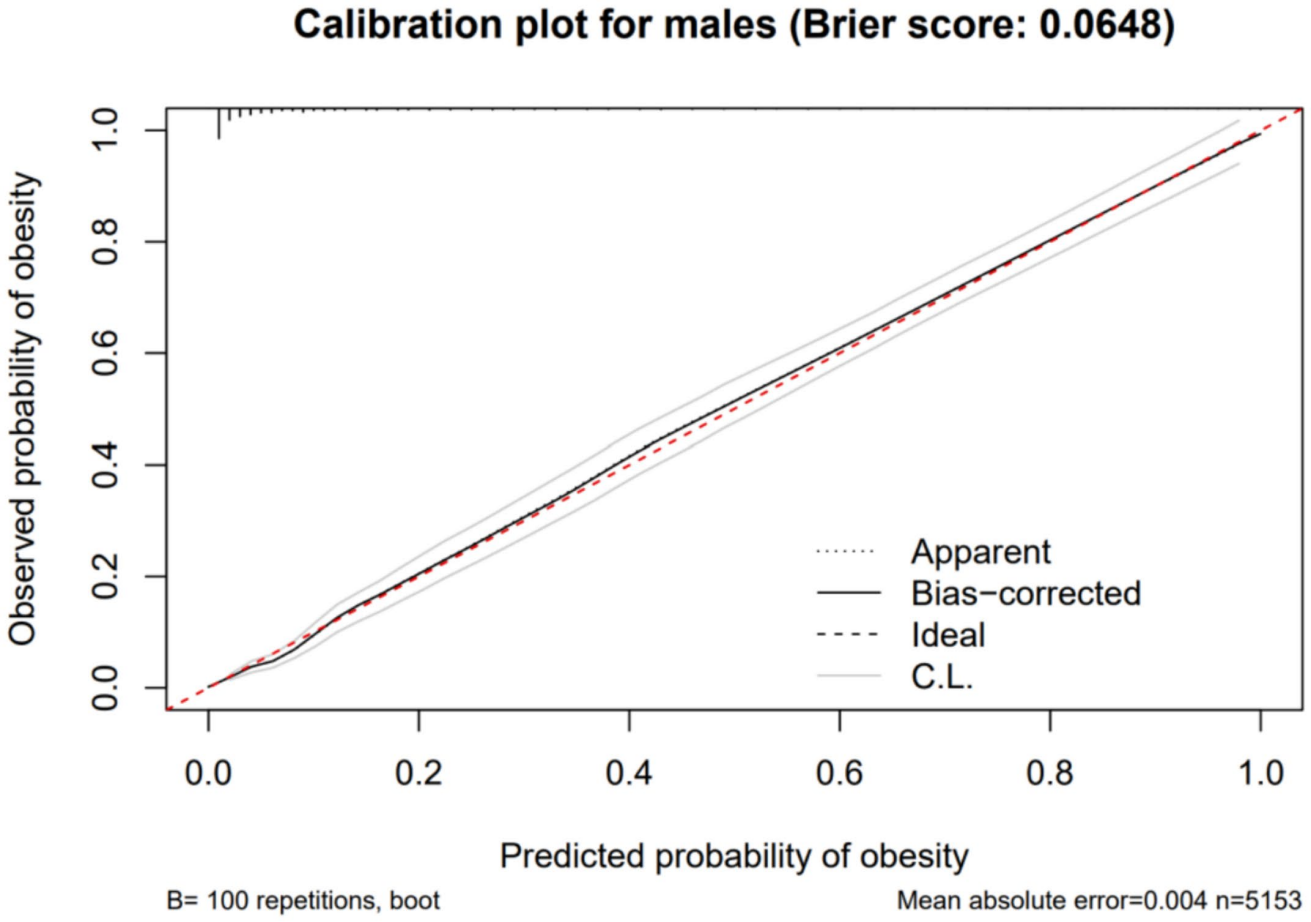
Calibration curve showing the expected rate of obesity predicted by neck circumference compared with the observed rate (with 95% CI) in Chinese young adult males, 2022–2024.

**Figure 7:**
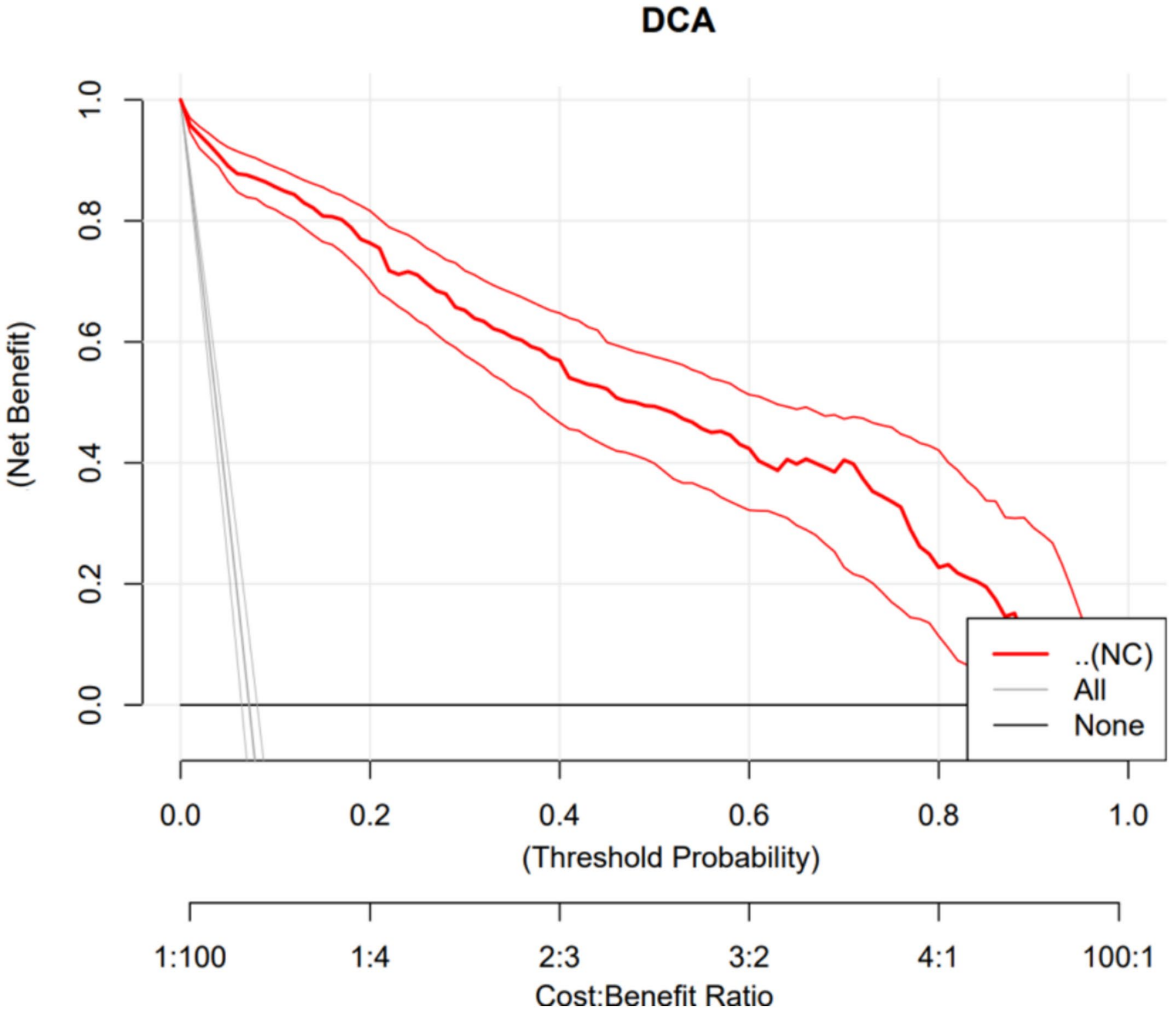
Decision curve analysis of a model using neck circumference (NC) to predict obesity in Chinese young adult females, 2022–2024.

**Figure 8:**
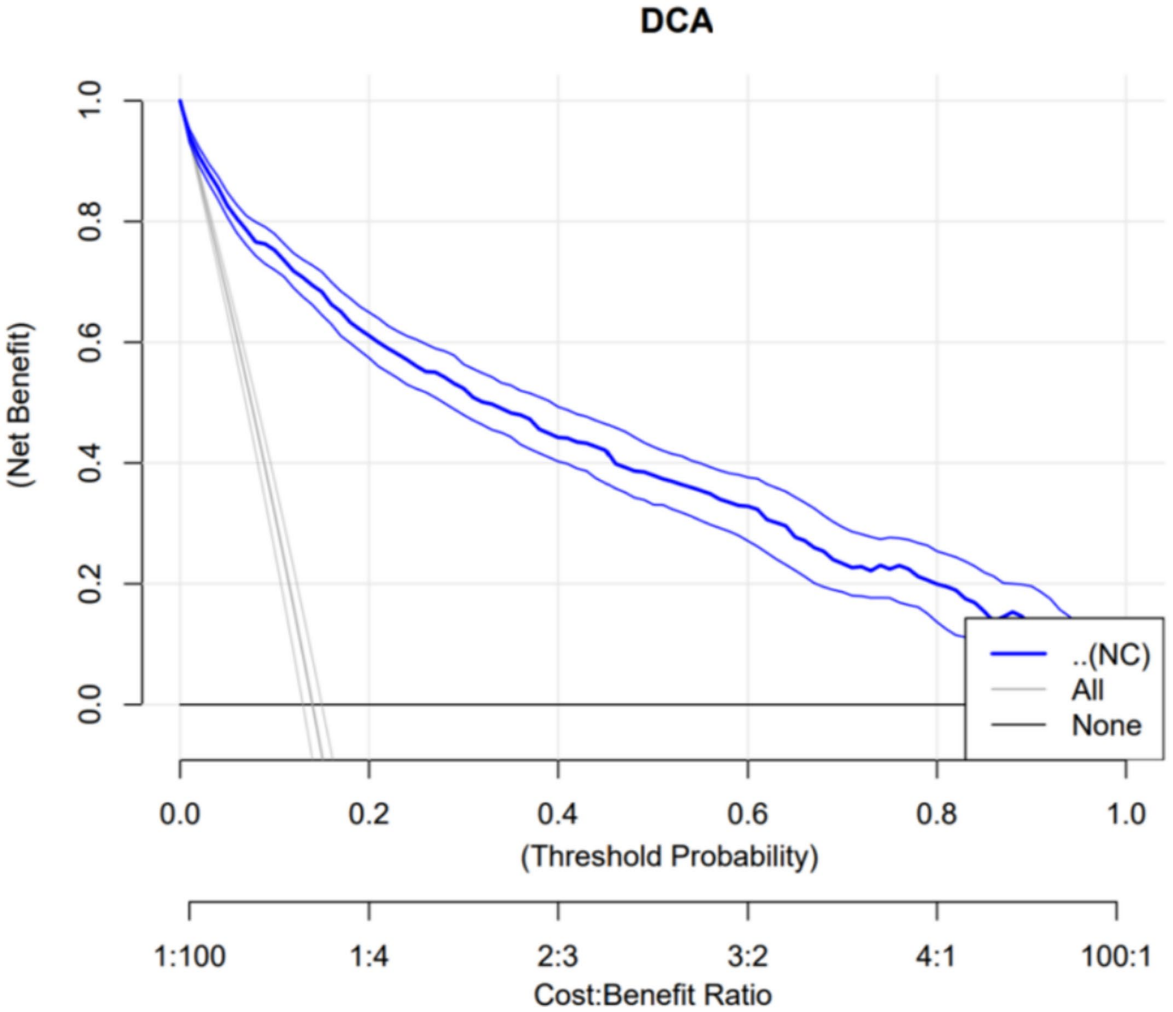
Decision curve analysis of a model using neck circumference (NC) to predict obesity in Chinese young adult males, 2022–2024.

Using obesity as the dependent variable, the ROC curves were plotted and analyzed for NC, VFA, PBF, and BMI by gender. See Figures 3, 4 and Table 3. In Figure 3, the areas under the ROC curves for NC, VFA, PBF, and BMI in females were 0.982, 0.976, 0.996, and 0.994, respectively (all *p* < 0.001), the area under the ROC curve for NC exceeded that of VFA, with an NC cut-off value of 36.25 cm, a sensitivity of 94.8%, and a specificity of 93.6%. Figure 4 presents the areas under the ROC curves for NC, VFA, PBF, and BMI in males, which were 0.918, 0.954, 0.985, and 0.979, respectively (all *p* < 0.001), in contrast to other obesity indicators, NC exhibited the smallest area under the ROC curve, with a cut-off value of 38.95 cm, a sensitivity of 83.8%, and a specificity of 85.5%. The calibration curve indicates that the Brier score is 0.0259 for females and 0.0648 for males, the results demonstrate that the female model has superior calibration, as shown in Figures 5, 6. The Decision Curve Analysis (DCA) results reveal that the net benefit of the obesity prediction model based on NC is significantly higher than that of the “all-treatment strategy” and the “no-treatment strategy,” as illustrated in Figures 7, 8. The consistency of obesity classification between neck circumference and BMI, as assessed by the Kappa test, showed: Kappa = 0.790 for females and Kappa = 0.644 for males (both *p* < 0.001), better consistency in females.

**Table 3:** Results of ROC curve analysis for predicting obesity in males and females using NC, VFA, PBF, and BMI in Chinese young adults, 2022–2024.

Sex	Variable	Cut-off point	AUC (95%*CI*)	Positive predictive value	Negative predictive value	Sensitivity (%)	Specificity (%)	Youden index	*p* value
Female	NC	36.25	0.982 (0.977, 0.987)	0.892	0.969	94.8	93.6	0.884	< 0.001
	VFA	101.84	0.976 (0.968, 0.984)	0.834	0.978	96.5	89.1	0.856	< 0.001
	PBF	39.33	0.994 (0.992, 0.996)	0.936	0.980	96.5	96.4	0.929	< 0.001
	BMI	28.135	0.996 (0.994, 0.998)	0.933	0.992	98.7	96.0	0.947	< 0.001
Male	NC	38.95	0.918 (0.907, 0.929)	0.766	0.904	83.8	85.5	0.693	< 0.001
	VFA	118.07	0.954 (0.947, 0.960)	0.837	0.917	86.0	90.5	0.765	< 0.001
	PBF	29.535	0.985 (0.982, 0.988)	0.854	0.979	96.6	90.7	0.873	< 0.001
	BMI	27.865	0.979 (0.975, 0.982)	0.839	0.970	95.1	89.7	0.848	< 0.001

NC, neck circumference; FMI, fat mass index; BMI, body mass index; PBF, percentage of body fat; VFA, visceral fat area; AUC, area under curve; PPV, positive predictive value; NPV, negative predictive Value.

## 4 Discussion

This study found that NC is significantly and positively correlated with BMI, PBF, FMI, and VFA. ROC curve analysis with obesity as the outcome variable revealed that in females, neck circumference outperformed VFA in predicting obesity; however, in males, its predictive efficacy was inferior to VFA, PBF, and BMI. The neck circumference cut-off values determined in this study were 38.95 cm for males and 36.25 cm for females, with corresponding sensitivities of 83.8 and 94.8%, and specificities of 85.5 and 93.6%, respectively, indicating that these cut-off values possess high diagnostic efficacy in both genders, with superior performance in females. Furthermore, based on the calibration curves and DCA curves, the AUC, sensitivity, specificity, classification consistency, and predictive accuracy of neck circumference in females were all excellent and significantly superior to those in males. Kappa test analysis revealed good consistency between neck circumference and BMI-based obesity classification (Kappa = 0.790 for females, Kappa = 0.644 for males), with better consistency observed in females. This indicates that neck circumference aligns more closely with the widely accepted BMI-based obesity criteria in young female populations, further highlighting its potential value in obesity screening. Due to the simplicity and speed of neck circumference measurement, which does not require complex equipment, it can be utilized as a potential screening tool for female obesity. In males, however, due to the limited predictive efficacy of neck circumference alone, it is necessary to conduct a comprehensive evaluation by combining it with other obesity indicators such as BMI, PBF, and VFA.

A cohort study involving 1,421 community individuals in China found that NC was independently associated with VFA, in which a 5% increase in NC was associated with an increase in VFA (27), a finding that is consistent with ours. Numerous studies have indicated that NC can serve as a simple and effective alternative to BMI for screening for obesity. Kiran et al. evaluated obesity based on NC among 282 medical students in India, identifying cut-off values of 37.1 cm for males and 31.4 cm for females (28). In a study of individuals aged 65 years and older in Chile, the cut-off values for NC to assess obesity were reported as 40.6 cm for males and 34.2 cm for females (29). The variation in cut-off values for assessing obesity based on NC across different populations may be partially attributable to the influence of age on the accuracy of these measurements. As individuals age, the metabolic capacity of neck soft tissues declines, collagen loss accelerates, superficial fat decreases, and fat accumulates in deeper regions (29). These age-related physiological changes lead to a alteration of the neck contour in the older adults, thereby influencing the cut-off values for obesity assessment based on NC. Additionally, the observed discrepancies across studies may arise from genetic and environmental factors, as well as differences in the diagnostic criteria for obesity. Iranian scholars have proposed a single NC cut-off value of 36.95 cm for both males and females (30), failing to account for differences in body composition between sexes. This oversight may lead to an increased rate of missed diagnoses of obesity in males; as evidenced by a sensitivity of 60%, which is significantly lower than that reported in the present study.

This study found that the predictive efficacy of NC for obesity demonstrates significant gender differences, which are likely attributable to variations in body composition, sex hormones, and adipose tissue distribution between males and females. Neck fat primarily consists of subcutaneous fat. Moreover, the correlation coefficient between subcutaneous adipose tissue and BMI is greater than that between subcutaneous adipose tissue and WC (13). This reveals that the association between neck circumference and BMI may be more pronounced in the female population, suggesting that neck circumference may have relatively higher potential for application in obesity screening among women, which is consistent with the findings of this study. Changes in NC during adulthood essentially reflect alterations in the amount of subcutaneous fat in the neck. Furthermore, females possess significantly more total subcutaneous fat in the anterior and posterior neck regions compared to males (31). Consequently, NC has insufficient sensitivity as a measure of generalized obesity in males. Furthermore, body composition and fat distribution differ between sexes, with females exhibiting a higher PBF, while males tend to have higher visceral fat levels (32). Sex hormones play a critical role in fat distribution; testosterone in males promotes an increase in muscle mass and a decrease in body fat, whereas estrogen in females encourages the accumulation of subcutaneous fat (33, 34). Additionally, females are more likely to store dietary free fatty acids in subcutaneous adipose tissue, whereas males tend to store them in visceral adipose tissue (35).

NC could serve as a valuable tool for screening people with obesity, particularly among women. From a public health perspective, NC assessment for obesity is highly practical, as it saves time for clinicians and researchers, thereby enhancing survey efficiency and participant throughput. This advantage is particularly relevant in specific populations, including pregnant women, athletes, and patients with ascites. Thus, NC may serve as a superior alternative indicator for obesity screening (29). Compared to WC and BMI, NC offers several unique advantages. Measuring NC requires only a soft tape measure, which is convenient for assessors to carry and use. In colder seasons, heavy clothing can compromise the accuracy of WC and BMI measurements, potentially leading to overestimation. Conversely, NC measurement is unaffected by the thickness of personal clothing, simplifying the process. Moreover, NC is not influenced by factors such as diet, respiration, or health conditions. For pregnant women, NC serves as a more effective indicator of obesity levels compared to WC and BMI. Implementing appropriate measures upon observing an increased NC can help mitigate the onset and progression of gestational diabetes and gestational hypertension. However, NC measurement is not recommended for individuals with conditions such as goiter or neck tumors, as it may lead to an overestimation of adiposity.

This study evaluated the correlation between NC and various body composition indicators, providing a novel perspective for obesity assessment. By utilizing FMI as the criterion for obesity evaluation, this approach mitigated the influence of confounding factors such as lean body mass and BMI. This effectively addresses the limitations of PBF in distinguishing fat distribution, thereby more accurately reflecting the degree of fat accumulation for obesity assessment. Moreover, the study has clarified that the predictive efficacy of neck circumference for obesity exhibits differences between males and females. In validating the potential value of neck circumference in assessing obesity, compared to studies that solely employ ROC curves, this research simultaneously incorporates calibration curves and DCA curves, further ensuring the reliability of the conclusions and highlighting its value as a screening tool for obesity in females, while also clarifying its relatively limited utility in males. However, this study has certain limitations: the collected samples were only from a young population in a single hospital, introducing selection bias, and it remains unclear whether neck circumference is an effective marker of body composition in older adults and patients with cardiometabolic diseases, affecting the generalizability of the study findings; this study is retrospective, limiting the inference of causality, and cannot establish a causal relationship between neck circumference and body composition indicators. Additionally, although DXA is an effective and widely used method for assessing body composition, future studies should consider using methods such as computed axial tomography or magnetic resonance imaging. Future research could conduct multicenter, large-sample prospective studies and collect clinical outcome indicators such as the occurrence of cardiovascular diseases and diabetes diagnosis, to refine the development of neck circumference as a diagnostic tool and provide more reliable evidence-based evidence for neck circumference as an indicator for assessing obesity diagnosis.

## 5 Conclusion

NC shows a robust correlation with key indicators of obesity, including BMI, VFA, PBF, and FMI. It can serve as a simple and effective screening tool for obesity in young females. However, for males, a comprehensive evaluation is necessary, which should combine various obesity indicators such as BMI, PBF, and VFA.

## Data Availability

The original contributions presented in the study are included in the article/Supplementary material, further inquiries can be directed to the corresponding author.
