# Supplementary material for: Relationship between neck circumference and body composition: a cross-sectional study based on a Chinese population
Source: Front Public Health. 2025 Nov 7;13:1693065. doi: 10.3389/fpubh.2025.1693065 (PMC12634340; doi:10.3389/fpubh.2025.1693065)
Supplement: Supplementary file 1 [file Data_Sheet_1.docx]

Supplementary Material

**Table S1** Sex-Specific Comparison of R² for Models of the Relationship Between Neck Circumference (NC) and Body Mass Index (BMI) in Chinese Young Adults, 2022–2024

| Sex | Linear Model R² | Quadratic Model R² | Logarithmic Model R² | Exponential Model R² |
| --- | --- | --- | --- | --- |
| Male | 0.652 | 0.653 | 0.637 | 0.637 |
| Female | 0.790 | 0.791 | 0.768 | 0.782 |

**Table S2** Sex-Specific Comparison of R² for Models of the Relationship Between Neck Circumference (NC) and Fat Mass Index (FMI) in Chinese Young Adults, 2022–2024

| Sex | Linear Model R² | Quadratic Model R² | Logarithmic Model R² | Exponential Model R² |
| --- | --- | --- | --- | --- |
| Male | 0.529 | 0.532 | 0.462 | 0.513 |
| Female | 0.688 | 0.695 | 0.576 | 0.675 |

**Table S3** Sex-Specific Comparison of R^2^ for Models of the Relationship Between Neck Circumference (NC) and Visceral Fat Area (VFA) in Chinese Young Adults, 2022–2024

| Sex | Linear Model R² | Quadratic Model R² | Logarithmic Model R² | Exponential Model R² |
| --- | --- | --- | --- | --- |
| Male | 0.456 | 0.469 | 0.370 | 0.446 |
| Female | 0.577 | 0.615 | 0.404 | 0.583 |

**Table S4** Sex-Specific Comparison of R² for Models of the Relationship Between Neck Circumference (NC) and Percentage of Body Fat (PBF) in Chinese Young Adults, 2022–2024

| Sex | Linear Model R² | Quadratic Model R² | Logarithmic Model R² | Exponential Model R² |
| --- | --- | --- | --- | --- |
| Male | 0.353 | 0.386 | 0.305 | 0.346 |
| Female | 0.467 | 0.580 | 0.357 | 0.469 |


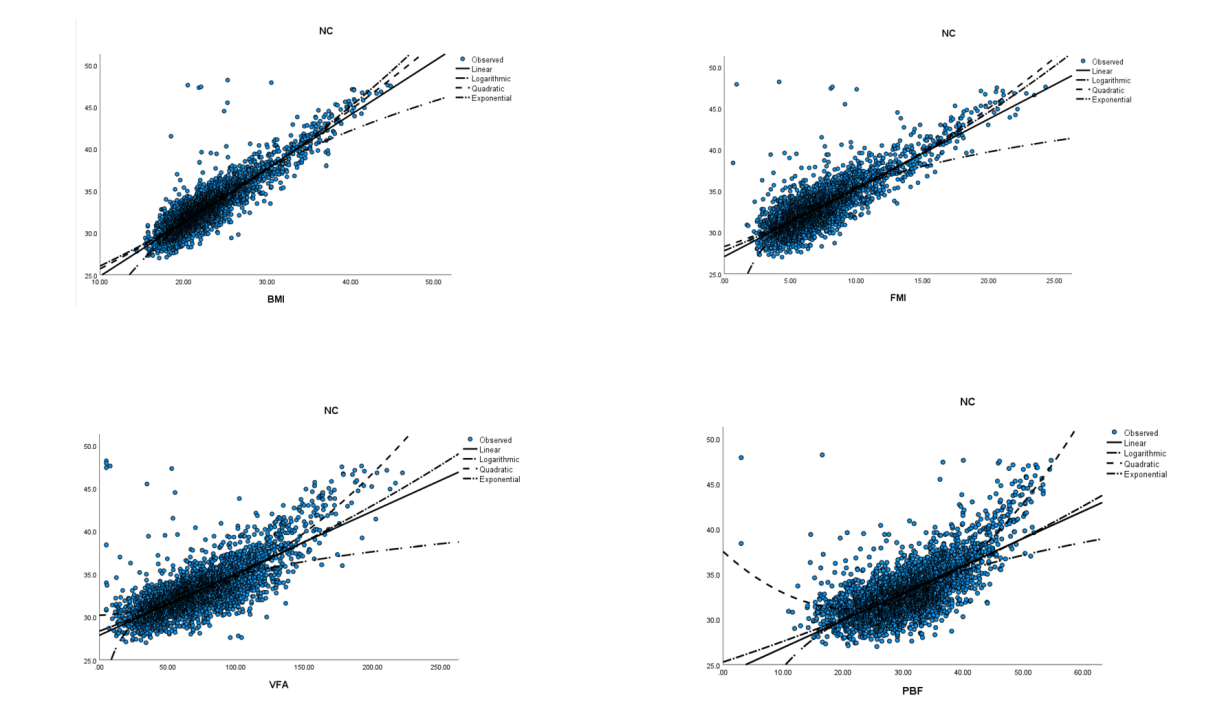


**Figure S1** Scatter Plots of Multi-Model Fitting Comparison Between Neck Circumference (NC) and Visceral Fat Area (VFA), Percentage of Body Fat (PBF), Fat Mass Index (FMI), and Body Mass Index (BMI) in Chinese Young Adult Females, 2022–2024

***Abbreviations:* NC** neck circumference; **FMI** Fat Mass Index; **BMI** body mass index; **PBF** Percentage of Body Fat; **VFA**: Visceral Fat Area


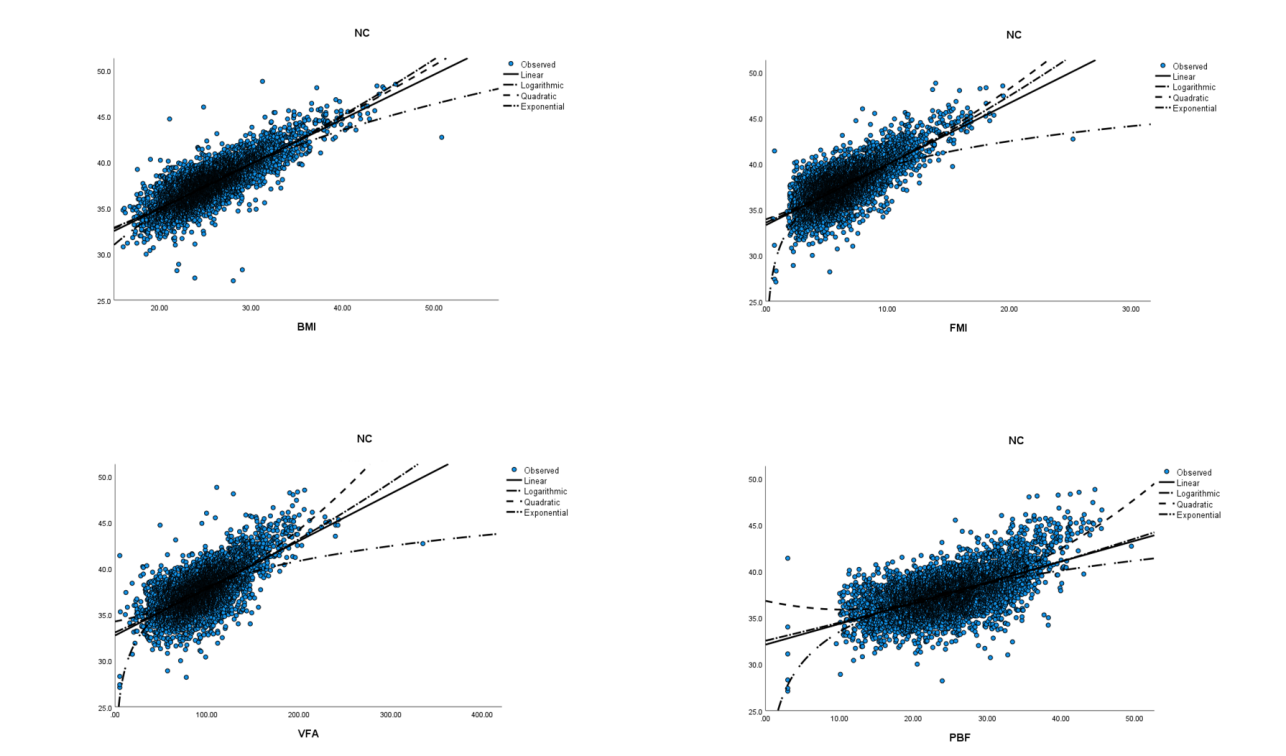


**Figure S2** Scatter Plots of Multi-Model Fitting Comparison Between Neck Circumference (NC) and Visceral Fat Area (VFA), Percentage of Body Fat (PBF), Fat Mass Index (FMI), and Body Mass Index (BMI) in Chinese Young Adult Males, 2022–2024

***Abbreviations:* NC** neck circumference; **FMI** Fat Mass Index; **BMI** body mass index; **PBF** Percentage of Body Fat; **VFA**: Visceral Fat Area


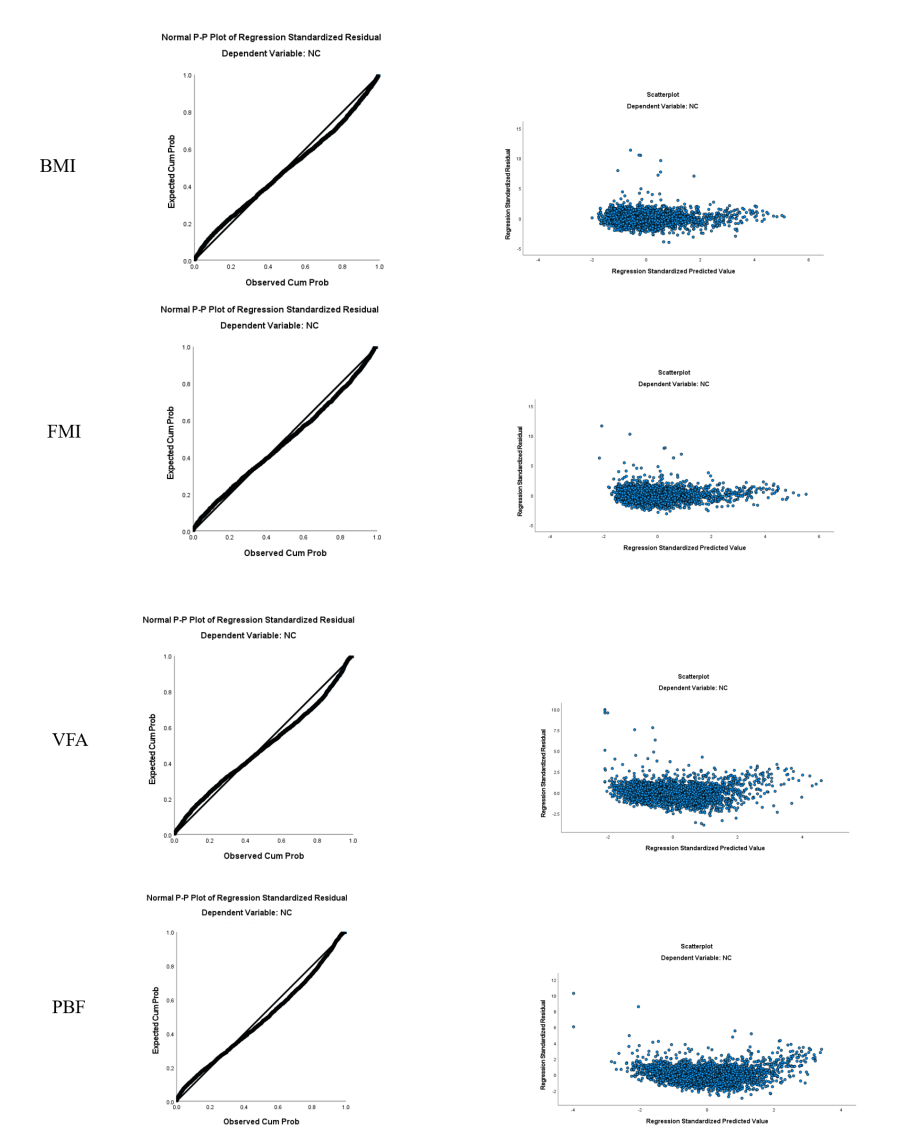


**Figure S3** Normal P-P plots of residuals and scatter plots of residuals versus predicted values for linear regression models between NC with VFA, PBF, FMI, and BMI in females

***Abbreviations:* NC** neck circumference; **FMI** Fat Mass Index; **BMI** body mass index; **PBF** Percentage of Body Fat; **VFA**: Visceral Fat Area


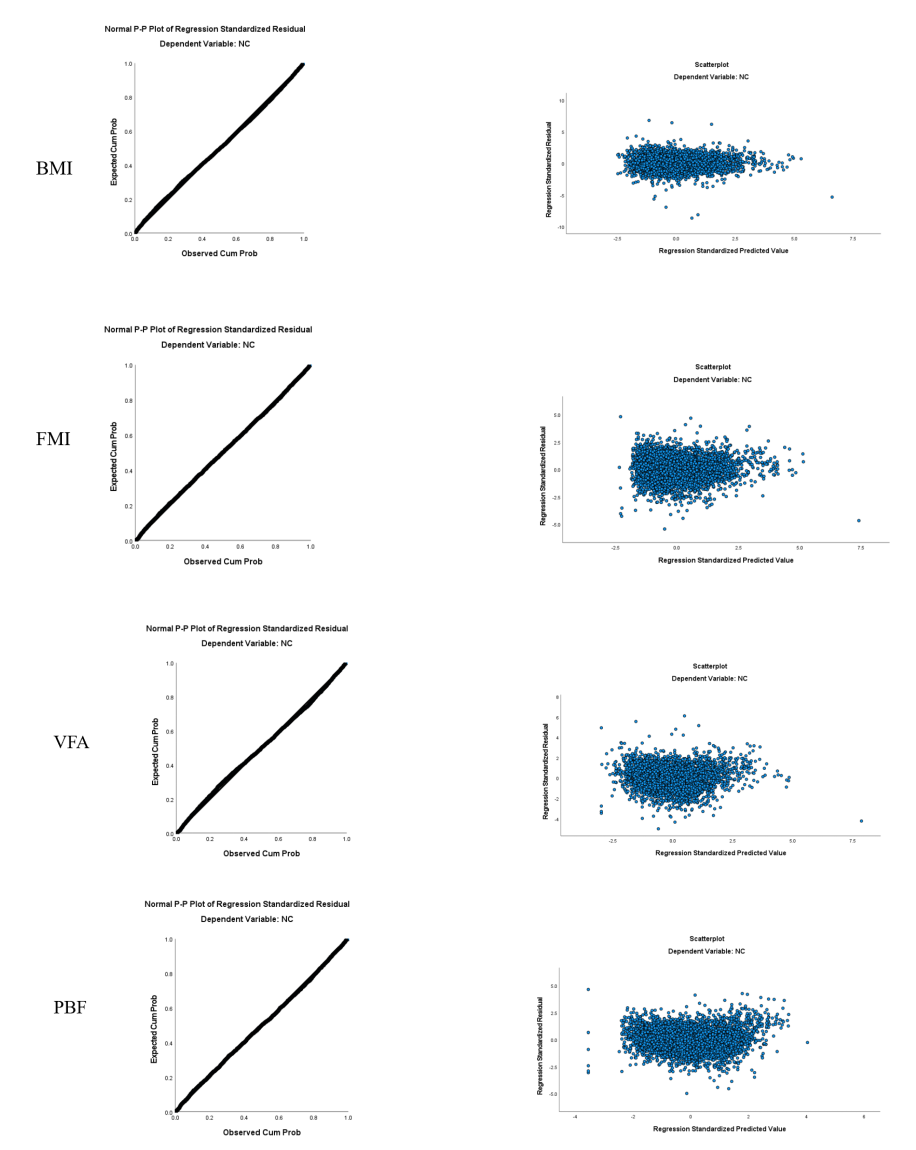


**Figure S4** Normal P-P plots of residuals and scatter plots of residuals versus predicted values for linear regression models between NC with VFA, PBF, FMI, and BMI in males

***Abbreviations:* NC** neck circumference; **FMI** Fat Mass Index; **BMI** body mass index; **PBF** Percentage of Body Fat; **VFA**: Visceral Fat Area
